# Lack of consensus in atypical congenital obstructive urethral lesions in children: Snapshot of the web-based ObsCUre (obstruction to the child urethra) study

**DOI:** 10.1097/CU9.0000000000000092

**Published:** 2022-08-02

**Authors:** Rachel Ng, Ahmed Adam, Nathan Poppleton, Christopher Oldmeadow, Aniruddh V. Deshpande

**Affiliations:** a Department of Paediatric Urology, John Hunter Children’s Hospital, New Lambton Heights, NSW, Australia; b Division of Urology, University of the Witwatersrand, Johannesburg, South Africa; c Klerksdorp Hospital, Klerksdorp, South Africa; d CREDITSS Service, Hunter Medical Research Institute, New Lambton Heights, NSW, Australia; e Priority Research Centre GrowupWell, Faculty of Health and Medicine, University of Newcastle, Callaghan, NSW, Australia

**Keywords:** Atypical urethral obstruction, Children, ObsCUre study, Urethral obstruction

## Abstract

**Background:**

Atypical Congenital Obstructive Urethral Lesions (ACOUL) are uncommon causes of urethral obstruction in children. They include Cobb’s collar or Moorman’s ring, Type III posterior urethral valve (PUV), congenital urethral narrowing and anterior urethral valves. This study is aimed to evaluate the knowledge and current practice amongst clinicians attending to ACOUL. An international online case based questionnaire was performed.

**Materials and methods:**

A survey was administered to members of international urological societies. It included 22 clinical questions on cases with ACOUL (14 questions suitable for statistical analysis) using cases of Type I PUV as controls. Two sets of paired questions evaluated change in opinion(s) after additional information was provided.

**Results:**

One hundred twenty-one participants responded with 71% reporting exposure of less than 5 cases per annum. In questions regarding diagnosis between 11.6% (14/121) and 21.5% (26/121) of participants identified the ACOUL as PUV. Among them, 66% of respondents agreed on ACOUL’s causative role in urethral obstruction. Gini coefficient was consistently lower for ACOUL compared to PUV: diagnosis (mean 0.33 vs. 0.44) and prognosis (0.23 vs. 0.43). High intra-rater concordance (kappa 0.420.57) was observed for paired questions–a mean of 5.79% (7.44% and 4.13% for questions 10 and 12, 16 and 17, respectively) of participants changed their answers from an alternate diagnosis to the correct diagnosis of ACOUL after viewing endoscopic images. High variation in management of ACOUL was noted (Gini 0.51).

**Conclusions:**

This global snapshot survey identified substantial inconsistency among clinicians dealing with ACOUL. Although rarely encountered in clinical practice, better overall education of ACOUL is warranted.

## 1. Introduction

**Figure 1:**
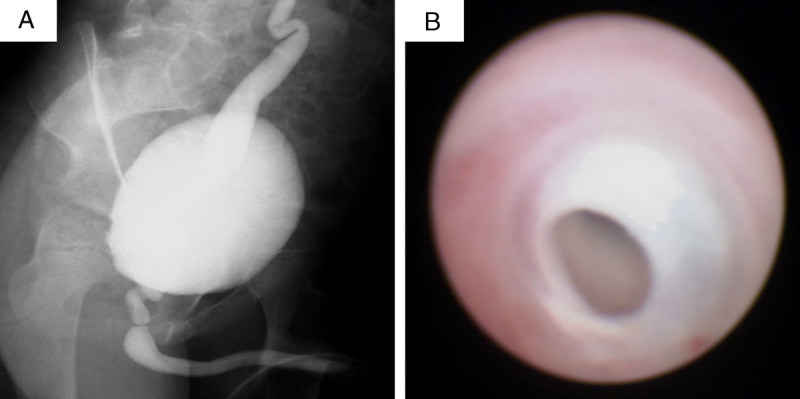
(A) Voiding cystourethrogram of Cobb’s collar, an atypical congenital posterior urethral narrowing, also showing vesicoureteric reflux; (B) Endoscopic (cystoscopic view) of the same patient as in (A).

Urethral obstructions other than type I posterior urethral valves (PUVs) are uncommon in children.^[1]^Current knowledge and practices around nomenclature, diagnosis, management, and follow-up appear poorly streamlined. A case in point is a recently encountered condition at our center, Cobb’s collar, also known as Moormann’s ring or congenital stricture (Fig. 1A and B), which had been initially reported decades ago, but no consensus definition or management algorithm has been established to date.^[2,3]^ Other terms used are Type III PUV, congenital obstructing posterior urethral membrane (COPUM), congenital urethral stricture, and atypical urethral narrowing. These conditions account for most but not all atypical obstructive urethral lesions in children. In order to reduce confusion, we have referred to them in this manuscript collectively as “atypical congenital obstructive urethral lesions” (ACOUL).

We were concerned that uncertainty around diagnostic definitions and a lack of clear management algorithms has resulted in significant clinical uncertainty and variation in clinical care. In modern practice, such variation in care is undesirable, as it can potentially reduce the quality and value of care through increased cost and/or increased morbidity associated with treatment. Although various congenital obstructive urethral lesions may appear similar anatomically, this does not necessarily mean that they will respond uniformly to the same management. Thus, if it is established that significant variation in clinical practice exists, further study can be undertaken to determine how best to proceed in creating consensus guidelines.

Hence, we conceived a survey titled “Obstruction to Child Urethra (ObsCUre)” to ascertain and objectively document the presence and possible magnitude of variation in practice related to ACOUL on a global scale. This survey was not aimed or designed to identify the best terminology or recommended

Furthermore, we aimed to assess variation in diagnosis, prognosis, and management options for ACOUL and examine how previous training experience, and additional information influence the ability of attending clinicians to diagnose and manage these conditions.

## 2. Material and methods

Survey participants were clinicians who practice pediatric urology and are current members of the surveyed urological societies. The following list of urological societies were invited to participate in the survey:

USANZ/SPUNZA (Urological Society of Australia and New Zealand/Society of Paediatric Urology of New Zealand and Australia)

SAUA (South African Urological Association)

ESPU (European Society for Paediatric Urology)

AAP (American Academy of Pediatrics)

CIPE (Associação Brasileira de Cirurgia Pediatrica *=* Brazilian Society of Pediatric Surgery)

SLAPS (Sri Lankan Association of Paediatric Surgeons)

BAPU (UK) British Association of Paediatric Urologists SIUP (Italy) Societa Italiana Urologia Pediatrica

MBPS (Mexico) The Mexican Board of Pediatric Surgery

SPU (Society for Pediatric Urology)

AUA (American Urological Association).

An invitation to complete an ethics-approved, web-based survey was subsequently sent electronically to members of societies mentioned above using email flyers and shared social media groups. Survey responses were collected from November 2017 until March 2018. The survey was created and hosted on SurveyMonkey (https://www.surveymonkey.com/r/2Z2S2JM), and took approximately 20 minutes to complete. The study did not utilize any private or public funding, and there was no incentive offered for completion of the survey.

### 2.1. Survey instrument and design

The survey (Supplementary File 1. ObsCUre Survey, http://links.lww.com/CURRUROL/A8) was developed by 3 investigators (AD, AA, and RN) and pilot tested on 2 pediatric urologists. The survey was based on recent cases of ACOUL and PUV managed by one of the investigators (AD). The pilot survey respondents provided feedback on survey content and design. The survey was then modified according to their feedback.

Approval was obtained from the Hunter New England Human Research Ethics Committee, Australia, Reference number 17/07/19/5.12.

### 2.2. Survey questions

The web-based survey was comprised of 28 structured questions (Supplementary File 1. ObsCUre Survey, http://links.lww.com/CURRUROL/A8). Question 1, as a preamble to the survey, asked for consent to participate in the survey, and question 28 asked for comments. Four questions ascertained basic demographic and society affiliation data, and the remaining 22 questions centered around ACOUL in children.

### 2.3. Clinical cases

The survey included details and images of 6 clinical cases, from which patient personally identifying information had been removed. The survey was designed with no option to return back to change answers to previously submitted questions in order that responses would more accurately reflect the clinical thinking of respondents. Two type I PUV cases were included to serve as controls for analysis.

Response data utilized for numerical analysis included questions about diagnosis (7), investigation (6) and treatment (1). Responses deemed inappropriate for numerical analysis included questions about diagnosis (1) and investigation (2) without a definitive correct answer, management questions with free-text responses (3), and clinical reasoning questions (2). The clinical reasoning questions asked specifically about the effects of previous training experience, and additional information on the clinician’s diagnostic and management abilities. All questions regarding management of cases in the survey included a choice “other” which allowed a free text response. This was designed to gain insight into the range of treatment options that respondents chose, and to allow them to provide their reasoning.

### 2.4. Demographics

The demographics section did not collect any personally identifiable information about clinicians.

### 2.5. Paired questioning

Questions 10 and 12, as well as questions 16 and 17, were linked questions. Participants were asked to provide a diagnosis in the first question (questions 10 and 16, respectively). Then, results of further investigations were given, and participants had the opportunity to change their original diagnosis in a subsequent question (questions 12 and 17, respectively). In order to assess views on prognosis, respondents were asked about the likelihood of a child developing bladder dysfunction secondary to PUV and ACOUL or similar diagnosis. This was designed to check for intra-rater concordance in diagnosis and prognosis by providing additional endoscopic information. We postulated that a significant proportion of respondents would alter their original responses in light of the new information (low intra-rater concordance).

### 2.6. Statistical analysis

Statistical analysis was performed using SAS v9.4 (SAS Institute, Cary, NC). Cohen and Fleisch kappa estimates were developed as appropriate for concordance and Gini indices for variance in responses.^[4]^ The higher the kappa coefficient, the greater the agreement. Gini coefficient is a measure of inequality in a distribution. A high Gini coefficient indicates that responses are relatively concentrated. A Gini of 0 indicates all answers are distributed equally. By inference, a low Gini coefficient suggests a lack of consensus. The maximum Gini coefficient for a multiplechoice question with n possible responses is given by (n-1)/n.^[5]^ We estimated Gini indices with and without missing responses and only reported those with missing responses, unless there was a substantial difference, in which case both were reported.

## 3. Results

### 3.1. Participant demographics

A total of 121 participants responded to the survey. Respondents included members of the following professional societies: ESPU, 20 (17%); SAUA, 18 (15%); SPUNZA, 13 (11%); other, 15 (12%); 55 respondents did not list any membership. The majority (71%) of respondents encountered less than 5 cases of urethral obstruction (other than PUV and meatal stenosis) per year. The median number of responses to each question was 75 (62%) with an interquartile range of 67–90 (Table 1).

### 3.2. Clinical cases

The median Gini coefficient for questions regarding the diagnosis of Cobb’s collar was 0.34, while the median Gini coefficient for the two included control questions about PUV was 0.44. When asked about the likelihood of bladder dysfunction, 82% of participants felt the likelihood to be significant in children with PUV (Gini coefficient 0.43), versus 35% for children with ACOUL (Gini coefficient 0.23). Upon further questioning about their response choice regarding prognosis of ACOUL, 45% of participants admitted their responses were based upon extrapolation of PUV data (Table 2).

### 3.3. Paired questions for intra-rater concordance

Two sets of paired questions (questions 10 and 12, 16 and 17) were included in the survey. In response to question 10, only 24.8% of respondents selected ACOUL or a similar diagnosis, and 21.5% responded likewise to question 16. After being shown cystour- ethroscopy images, a mean of 5.79% of respondents (7.44% for question 12, and 4.13% for question 17) revised their original diagnosis to the correct diagnosis of ACOUL. For the first set of paired questions, fewer respondents correctly diagnosed ACOUL after seeing the endoscopic images (24.0%) than did in response to the original question (24.8%). Whereas for the second set of paired questions, 4.1% more respondents correctly diagnosed ACOUL after seeing endoscopic images (25.6%) compared to the original question (21.5%). In contrast, a mean of 54.6% of respondents maintained an incorrect answer for both sets of paired questions. The overall kappa coefficient was 0.56 (95% confidence interval, 0.46–0.66) and 0.57 (95% confidence interval, 0.46–0.68) between questions 10 and 12 and questions 16 and 17, respectively (Table 2).

### 3.4. Previous training or experience

When asked about their choice of treatment, 37% of respondents replied they had “seen it once or twice before in a child and think it is best way [to treat],” while 24% could not recall being taught about ACOUL or similar uncommon conditions and were unsure of the best treatment.

Selected comments from participants included: *“Challenging cases. Would love to know the correct answers. Made me feel like I was sitting final exams”; “I want to receive results and the paper. I want to continue learning about those rare conditions. Thanks and congratulations.”; “Good luck!! Important issue! More history for the boy with the bulbar ring (COPUM / Stricture) would be interesting. Looks acquired—but in an infant?”;“Great work! Could we have the answers at the end of the survey?”;“Very interesting survey”* and *“Congratulations for the idea.”*

## 4. Discussion

In a first survey of its kind on ACOUL or similar uncommon conditions in endoscopic pediatric urology, this survey has identified significant variability in respondents’ perceptions about the diagnosis, prognosis, and management of these conditions. Furthermore, over three quarters of respondents reported that their perceptions stemmed from educated guesswork or extrapolation from data for a different urological condition. Given that this survey sample captured a cross section of members from various national urological societies, survey responses raise the possibility of unnecessary clinical variability and associated effects on healthcare expenditures. Such variability further emphasizes the need for urgent standardization of clinical care relating to this topic, notwithstanding the admitted methodological weaknesses of any survey.

In 1993, Dewan et al. challenged Young’s classifications by suggesting that most posterior urethral obstructions fall under a unifying morphological diagnosis, for which the term COPUM was proposed.^[6,7]^

The diagnoses Cobb’s collar, Moorman’s ring, congenital urethral stricture, COPUM, and atypical urethral narrowing were developed decades ago, and since then no significant effort has been made to standardize diagnostic criteria. For the purposes of this survey, we considered these conditions synonymous with ACOUL as a preliminary step toward standardization. As explained above, ACOUL is a rare condition, which is a significant contributing factor to the lack of evidence regarding diagnosis and treatment. In retrospect, we noted that this survey could be considered an example of how modern technology could be used to share concepts and data between research centers so that more robust data about rare conditions could be collected and analyzed.

### 4.1. Terminology and diagnosis

Congenital urethral obstructions in boys are most commonly caused by Type I PUV as described by Young.^[1]^ Young also described Type III PUV as a circular membrane or diaphragm in the bulbar urethra, with a perforation in the center and oriented transversely to the urethral axis.^[1,8]^ This obstruction was able to be visualized on urethrocystoscopy and independent of the verumontanum.^[1]^ In 1968, Cobb et al. reported a series of similar congenital strictures, and the name “Cobb’s collar” was adopted for this abnormality. Its embryological origin was postulated to be a persistent urogenital membrane.^[2]^ Meanwhile, Moorman detailed a similar stenosis of the bulbar urethra, and the term often used in Europe was “Moorman’s ring.”^[3]^ Cobb’s collar is sometimes called a bulbar urethral obstruction, but it is almost always discussed with other posterior urethral obstructions.^[9]^

### 4.2. Pathology and clinical correlation

Some authors have cast doubt on the actual significance of stenosis or obstruction caused by ACOUL. Several papers have pointed out that it is difficult to claim that Cobb’s collar was the causative pathology underlying the patients’ urinary symptoms in the original report.^[9–11]^ Dewan et al. claimed that even if Cobb’s collar was found on urethrocystoscopy, if it was not seen on voiding cystourethrography then it was not of pathological importance.^[11]^ In our study, 66% of respondents believed that ACOUL could be the cause of the symptoms presented in the respective cases within the survey (Supplementary File 1. ObsCUre Survey, http://links.lww.com/CURRUROL/A8). Others ascribed the symptoms to functional causes such as dysfunctional voiding. We see this discrepancy as indicating a high level of uncertainty that needs to be further evaluated objectively, since it has direct treatment implications.

### 4.3. Variation in diagnosis, prognosis, and management

The general lack of consensus in the literature regarding ACOUL was reflected in the findings of our study. There was limited agreement on “index of suspicion” of ACOUL as the cause of symptoms presented in patient cases, as mentioned above. Furthermore, the Gini coefficient for the diagnosis of ACOUL was low (median 0.34), which indicates a higher degree of variability in responses as compared to the designated control diagnosis PUV (median 0.44). This difference may stem from various reasons and factors which have been superficially explored in this survey, but more importantly points to the need for further study and urgent standardization.

The paired questions produced a moderately high intra-rater level of agreement.^[4]^ This would suggest that participants were relatively “fixed” on their original answers and were not influenced by the addition of cystourethroscopy images. These are somewhat counterintuitive findings. Given the rarity of the condition and likely imprecision in the provisional diagnoses, one would expect the participants to alter them with additional information; however this did not appear to be the case.

Regarding prognosis, 82% of respondents deemed the likelihood of bladder dysfunction significant for children with PUV (Gini coefficient 0.43) versus only 35% for children with ACOUL (Gini coefficient 0.23). This highlights a perceived difference in severity between the two pathologies with participants’ responses suggesting that PUV is associated with a higher prevalence of bladder dysfunction. Concerningly perhaps 45% of participants reported basing their answers about the prognosis of ACOUL on extrapolation of data on PUV, while 34% said their answer was based on an educated guess. This should confirm the need for consensus-based guidelines specifically for ACOUL and similar conditions.

The questions with options for free text responses regarding ACOUL management demonstrated a clear lack of consensus that the authors feel reflects the lack of consensus guidelines. For instance, 81 free-text responses were given for question 8 (regarding management of possible ACOUL), ranging from nonspecific statements such as “cystoscopy” to specific medications alone.

The aims of this survey did not include determining the preferred treatment for ACOUL, and statistical analysis was not conducted for certain questions, given the variability of free text responses. However, the broad range of responses provides evidence that significant differences in management exist, which should be addressed by further investigation. An initial step might be to clarify diagnostic criteria, which could serve as a foundation upon which investigations into management could be based.

Perhaps one of the most important causes of lack of consensus in international practice is a lack of robust data. Further substantiating data for this argument lies in the high-level credentials of our participants (Table 1). When asked directly, 79% of participants said that they adopted a “best guess” approach or used extrapolation of knowledge about PUV in answering questions about the prognosis of Cobb’s collar, as opposed to relying on robust literature. One possible solution to the issue of limited data is further collaboration between research centers; in this modern age, new information technologies could make such collaboration much more convenient.

There was no consistency within societies within the same geographic region.

We observed uniform heterogeneity in diagnostic criteria and management recommendations within the 3 societies that had a relatively large sample size response (SAUA (29), SPUNZA (13) and ESPU (25)). The variation in responses of an index case study, which we felt was most representative, was equally distributed within and without the societies and the overall cohort.

Another condition fraught with confusion in nomenclature in pediatric urology is the dismembered pyeloplasty operation for pelvi-ureteric junction obstruction. Our previous attempt to reclassify the actual incision performed has now set the stage for accurately describing the surgical approach utilized,^[12]^ and an expert “Round Table” discussion has attempted to resolve other conundrums regarding pelvi-ureteric junction obstruction management.^[13]^ Now that this study has highlighted significant variance in clinical practice regarding ACOUL, discussion surrounding the standardization of management can begin.

**Table 1:** Tabulation breakdown of participant credentials.

Characteristic	Number (%)
Affiliation with societies	
Not entered	55 (45)
ESPU	20 (17)
Other	15 (12)
SAUA	18 (15)
SPUNZA	13(11)
Primary work sector	
Academic/university centre	29 (43)
Private Practice	14 (21)
Both but predominantly in an academic/university centre	17 (25)
Both but predominantly in private practice	7(10)
Case load (number of urethral obstruction cases other than type I PUV and meatal stenosis seen in scope of practice over 12 month period)	
Not entered	55 (45)
Less than 5 cases	17 (14)
More than 5 cases but less than 20 cases	47 (39)
More than 20 cases	2 (2)

ESPU = European Society for Paediatric Urology; PUV = posterior urethral valve; SAUA = South African Urological Association; SPUNZA= Society of Paediatric Urology of New Zealand and Australia.

**Table 2:** Measures of agreement and variation between participant responses.

Question theme	No. salient responses (%)		Intra-rater kappa (between questions)	Gini coefficient (low Gini = high variability)	Maximum Gini
**Diagnosis**				
Index of suspicion for urethral obstruction (Q 5,21)	(no + unsure) = 34%			0.33, 0.40	0.8
Diagnosis of ACOUL (Q 7,10,12,16,17)				Median 0.34	0.8
				Range (0.27–0.40)	
Diagnosis of PUV (control questions)				0.57	0.8
**Linked questions 10 and 12: Diagnosis of ACOUL or similar**							
Question 10 (before urethroscopy image)	ACOUL or similar 30 (24.8)			0.28	0.8
Question 12 (after urethroscopy image)	ACOUL or similar 29 (24)		0.56 (high)	0.3	0.8
**Linked questions 16 and 17: Diagnosis of ACOUL or similar**							
Question 16 (before urethroscopy image)	ACOUL or similar 26 (21.5)			0.23	0.8
Question 17 (after urethroscopy image)	ACOUL or similar 31 (25.6)		0.57 (high)	0.33	0.8
Prognosis: No. of responses (%)				
Likelihood of bladder dysfunction in PUV	significant likelihood 60 (82)			0.43	0.67
Likelihood of bladder dysfunction in ACOUL or similar	significant likelihood 25 (35)			0.23	0.67
**Reasons for choice of answer for prognosis of ACOUL or similar (no. of responses [%])**							
Reasons	educated guess 24 (34)
	extrapolation of PUV data 32 (45)			
	guidance by mentor 7 (10) robust evidence available 8 (11)			0.32	0.75
**Reasons for choice of treatment of ACOUL or similar (no. of responses [%])**							
Reasons	cannot remember being taught 19 (24)
	have seen it before and think it is best treatment option 29 (37)			
	have been taught and am confident 15 (19) am certain 15 (19)			0.15	0.75

ACOUL = atypical congenital obstructive urethral lesions; PUV =posterior urethral valve.

### 4.4. Limitations

We acknowledge this study was limited by the difficulty of reproducing a detailed complete clinical context in a case-based scenario questionnaire, which may have influenced the way respondents interpreted each case. We also received comments regarding respondents’ concerns about the quantitative nature of some questions and format of information presented. These points are an accepted limitation of the survey instrument used. Additionally, despite our efforts to survey a representative crosssectional sample of organizations all over the world—including Europe, South Africa, the USA, Australia, and New Zealand— that would provide information about up-to-date clinical practice globally, we cannot claim that we have surveyed a truly “global” cross section of urological societies.

## 5. Conclusions

This survey, which represents a broad cross-section of members of urological societies, identifies significant uncertainty and variations among clinicians regarding the diagnosis, investigation, prognosis, and management of atypical posterior urethral “narrowings” in boys. This survey also confirms the lack of reference resources and, hence, the need for structured guidelines and management algorithms for use in the management of this condition. We have proposed an umbrella term “atypical congenital obstructive urethral lesions” (ACOUL) for these atypical posterior urethral “narrowings,” intended to be used as a starting point in clarifying the diagnosis of these conditions. However, we admit that much more clarification regarding the definition of this nomenclature is warranted with directed future studies.
